# Sustainable Utilization of Coffee Pulp, a By-Product of Coffee Production: Effects on Metabolic Syndrome in Fructose-Fed Rats

**DOI:** 10.3390/antiox14030266

**Published:** 2025-02-25

**Authors:** Nelson Andrade, Ilda Rodrigues, Francisca Carmo, Gabriela Campanher, Isabella Bracchi, Joanne Lopes, Emília Patrício, João T. Guimarães, Juliana A. Barreto-Peixoto, Anabela S. G. Costa, Liliana Espírito Santo, Marlene Machado, Thiago F. Soares, Susana Machado, Maria Beatriz P. P. Oliveira, Rita C. Alves, Fátima Martel, Cláudia Silva

**Affiliations:** 1 Laboratório Associado para a Química Verde—Tecnologias e Processos Limpos (REQUIMTE/LAQV), Department of Chemical Sciences, Faculty of Pharmacy, University of Porto, 4050-313 Porto, Portugal; nandrade@med.up.pt (N.A.); jpeixoto@ff.up.pt (J.A.B.-P.); acosta@ff.up.pt (A.S.G.C.); lsanto@ff.up.pt (L.E.S.); mcmachado@ff.up.pt (M.M.); up201902664@up.pt (T.F.S.); smachado@ff.up.pt (S.M.); beatoliv@ff.up.pt (M.B.P.P.O.); rcalves@ff.up.pt (R.C.A.); claudiasilva@med.up.pt (C.S.); 2 Unit of Biochemistry, Department of Biomedicine, Faculty of Medicine of Porto, University of Porto, 4200-465 Porto, Portugal; irodrigues@med.up.pt (I.R.); up201606420@up.pt (F.C.); gabi.campanher@hotmail.com (G.C.); ibc@ess.ipp.pt (I.B.); jtguimar@med.up.pt (J.T.G.); 3 Instituto de Investigação e Inovação em Saúde (i3S), University of Porto, 4200-465 Porto, Portugal; 4 School of Medical Sciences, University of Örebro, Campus USÖ, S-701 82 Örebro, Sweden; 5 Department of Pathology, São João Hospital Centre, 4200-319 Porto, Portugal; u007769@chsj.min-saude.pt (J.L.); u003974@ulssjoao.min-saude.pt (E.P.); 6 Department of Clinical Pathology, São João Hospital Centre, 4200-319 Porto, Portugal

**Keywords:** coffee pulp, metabolic syndrome, fructose, liver, adipose tissue

## Abstract

Metabolic syndrome (MetS) is a cluster of metabolic abnormalities that include insulin resistance, impaired glucose tolerance, dyslipidemia, hypertension, and abdominal obesity. Coffee production generates large quantities of waste products, which pose a serious threat to the environment. However, coffee by-products, such as coffee pulp (CP), possess an undeniable wealth of bioactive components. Based on this, we investigated whether a 10-week dietary intervention with 250 mg/kg/d of CP could prevent or ameliorate MetS in high-fructose-fed rats. Consumption of CP by rats fed a high-fructose diet reduced body weight gain, lowered systolic blood pressure (SBP), fasting plasma glucose and insulin levels, and improved insulin resistance compared to rats fed a high-fructose diet alone. At the hepatic level, CP attenuated the increase in lipid storage, reduced lipid peroxidation, and improved glutathione levels when combined with a high-fructose diet. CP also affected the expression of key genes related to glucose and lipid metabolism in hepatic and adipose tissues, in rats fed a fructose-rich diet. This study demonstrates that CP ameliorates several consequences of high-fructose-induced MetS in the rat (weight gain, hypertension, glucose intolerance, insulin resistance, changes in liver, and adipose tissue function). Hence, our data provide evidence that CP consumption in the context of a high-fructose diet can be used to improve MetS management.

## 1. Introduction

Metabolic syndrome (MetS) is recognized as a cluster of metabolic dysregulations that includes insulin resistance, hyperglycemia, hypertension, dyslipidemia, and central obesity [1,2]. MetS is a pro-inflammatory state caused by abnormal glucose metabolism, which leads to elevated type 2 diabetes (T2D), cardiovascular disease, and cancer risk [1,2]. The predisposition to MetS is influenced by complex interactions between genetic factors and diet [3], and this condition has gained considerable relevance as the global obesity epidemic has grown exponentially [1]. Dietary habits and physical exercise are considered the primary preventive interventions of the MetS [3,4] and, in this sense, functional foods have gained a lot of interest lately, as they have been shown to present benefic effects on MetS [4].

In this context, coffee has garnered special attention because of its potential impact on metabolism [5,6,7]. Due to its organoleptic and psychoactive properties, coffee is one of the most consumed beverages in the world [8,9]. Currently, more than 500 billion cups of coffee are consumed each year [10]. However, coffee manufacturing produces large amounts of by-products with an undeniable richness in bioactive components, which are discarded every year around the world, leading to serious environmental concerns [8,11]. Coffee pulp (CP) (the outer part of the coffee cherry) is the first by-product of the coffee wet post-harvest processing, and represents approximately 40 to 50% of the coffee fresh berry weight [12,13]. CP has been reported to be composed (in dry weight) of carbohydrates (58 to 85%), reducing sugars (14%), proteins (8 to 11%), minerals (3 to 7%), lipids (0.5 to 3%), tannins (5%), and caffeine (1%) [13,14]. CP is also known for its high content of phenolic compounds, such as chlorogenic acids (62 mg/g), caffeic acid (16 mg/g), and gallic acid (3 mg/g) [15,16]. These concentrations vary widely according to the geographical origin, and extraction and drying methods used. Of note, coffee pulp/husk is authorized as a new food under the EU Regulation (EU) 2015/2283 [17].

Our research group has investigated the chemical and bioactive composition of different by-products, including CP, obtained along the coffee production chain. In a previous study, we highlighted arabica coffee pulp as a valuable source of minerals, protein, dietary fiber, antioxidants, and caffeine, while also presenting a low-fat content [8].

Research on the possible health benefits of coffee has focused on roasted coffee as a beverage rather than on intermediary products, such as CP. However, as mentioned, CP presents a high content of dietary fiber [8], which is known to lower cholesterol levels, control blood sugar levels, and reduce the risk of excessive weight gain, obesity, and T2D [18,19]. Moreover, CP presents also significant amounts of caffeine and chlorogenic acids [8], which also regulate lipid and glucose metabolism, preventing chronic diseases, such as T2D and obesity [20]. So, it is anticipated that adding CP to the diet might help to reverse the metabolic dysregulations found in MetS, while also providing extra value to coffee growers as a current waste product. Notwithstanding, preliminary studies have shown that CP has comparable effects to roasted coffee products on metabolic pathophysiological processes. Namely, CP extracts exhibit in vitro antioxidant activity [8,21,22], hypolipidemic [23] and anti-inflammatory properties [24], and is able, in vivo, to reduce intestinal cholesterol absorption [25], ameliorate liver steatosis [26], normalize glucose homeostasis, improve plasma lipid profile [27,28], and contribute to reduced obesity, dyslipidemia, and hyperglycemia in high-fat diet-fed rodents [28].

Modern diets are characterized by a high intake of fructose (e.g., sucrose and high-fructose corn syrup) and the consumption of this sugar is associated with MetS development [29], both in rodent experimental models and human populational studies [29,30]. So, it is crucial to understand the mechanisms involved in the nefarious metabolic consequences of fructose intake and to develop new interventions to reduce its consequences. In this context, this study aimed to evaluate if a dietary intervention with CP ameliorates MetS markers in high-fructose-fed rats. We assessed various metabolic parameters related to MetS in vivo (body weight, body length, abdominal circumference, glycemia, and blood pressure) and the functionality and structure of insulin-sensitive organs (liver and mesenteric adipose tissue) was assessed ex vivo by the histological examination and assessment of key genes related to glucose and lipid metabolism.

## 2. Materials and Methods

### 2.1. Coffee Pulp Sample Preparation

Dried coffee pulp from Costa Rica (Alajuela region) was acquired from the coffee roaster company TADAH kafferosteri AB (Lenhovda, Sweden). The sample (~1 kg) was ground using a Grindomix GM 200 miller (Retsch, Haan, Germany) and then manually homogenized. After that, the ground homogenized powder was divided in small parts, stored in vacuum, and was kept protected from light and at room temperature until the analyses and the in vivo assays were performed.

### 2.2. Coffee Pulp Nutritional Analysis

Moisture content was determined in an infrared moisture analyzer (105 °C) (DBS-KERN & SOHN GmbH, Balingen, Germany). Total and insoluble dietary fiber, total ash, total protein, and total fat were analyzed using standard normalized methods [31]. Soluble fiber and available carbohydrates were calculated by difference.

For fatty acids analysis, the lipid fraction was extracted as described [32]. Very briefly, the lipid fraction of the sample (150 mg) was extracted with absolute ethanol and n-hexane, followed by a salting out step with NaCl (1%, *m*/*v*). After centrifugation, the upper layer was collected, and the residue was re-extracted twice with n-hexane. The combined organic phases were mixed with anhydrous Na_2_SO_4_ and after a new centrifugation, the supernatant was collected and taken to dry under a nitrogen stream. Fatty acids were then derivatized into methyl esters, according to ISO 12966-2:2017 [33] and analyzed in a GC-FID system following the instructions of [34].

Caffeine and chlorogenic acids levels were determined according to [8] with minor modifications. In brief, the sample (20 mg) was extracted with 900 µL of water/ethanol (1:1) for 60 min. After centrifugation, the supernatant was collected, and the residue was re-extracted (30 min). The combined supernatants were analyzed by HPLC with diode array detection, following the conditions described by [8].

### 2.3. In Vivo Experiments

#### 2.3.1. Animals

Twenty-four male adult (7 weeks-old) Sprague-Dawley rats (250–315 g body weight) were obtained from Charles River Laboratories (Saint Germain Nuelles, France). Upon arrival, rats were housed in pairs, in a temperature-controlled (20–22 °C) room on a 12:12 h dark–light cycle. Before the experimental procedure, animals were allowed an acclimation period of 7 days in the environment with free access to standard laboratory pellet chow (diet #4RF21 certificate, Mucedola, Milan, Italy) and tap water. Animals were handled and cared for according to the Council of Europe guidelines for the use of laboratory animals (86/609/EEC) and Law 129/92. The experimental protocol was approved by the Animal Welfare and Ethics Committee (ORBEA) of the Faculty of Medicine of the University of Porto, Portugal, and by the Directorate General of Food and Veterinary of the Portuguese Government (0421/000/000/2022).

#### 2.3.2. Fructose-Induced Metabolic Syndrome

MetS was induced in normoglycemic rats by replacing tap water with D-fructose (VWR International, Radnor, PA, USA) 20% (*w*/*v*) in tap water for 10 weeks. Fructose solution was freshly prepared every alternate day and administered ad libitum.

#### 2.3.3. Experimental Design

**Figure 1:**
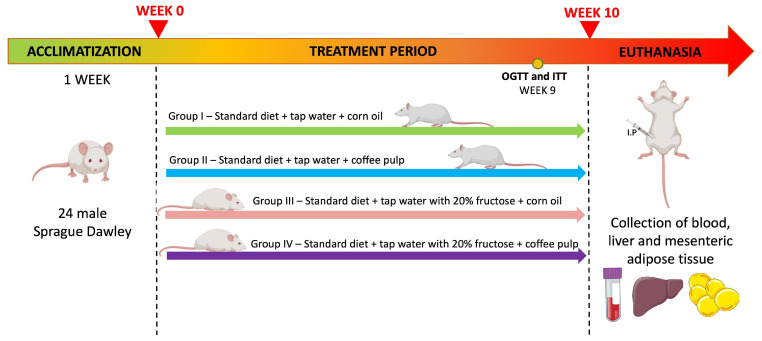
Experimental design. Abbreviations: OGTT, oral glucose tolerance test; ITT, insulin tolerance test.

As depicted in Figure 1, the twenty-four male rats were randomly divided into four groups, with each group comprising six animals, as follows: (I) Control (CONT)—this group received ad libitum standard diet and tap water and 0.25 mL/kg of corn oil daily (by gavage); (II) Coffee pulp (CP)—this group received ad libitum standard diet, tap water, and a daily dose of CP (250 mg/kg mixed in 0.25 mL/kg of corn oil daily; by gavage); (III) fructose (FRUCT)—this group received ad libitum standard diet and tap water supplemented with 20% (*w*/*v*) fructose and 0.25 mL/kg of corn oil daily (by gavage); and (IV) fructose + coffee pulp (FRUCT + CP)—this group received ad libitum standard diet and tap water supplemented with 20% (*w*/*v*) fructose and a daily dose of CP (250 mg/kg mixed in 0.25 mL/kg of corn oil; by gavage). All experimental groups were fed with the standard laboratory chow diet (diet #4RF21 certificate, Mucedola, Milan, Italy). The chemical composition of the feed was determined as described for CP (Section 3.1) (Supplementary Table S1).

Various metabolic syndrome-related parameters were measured weekly throughout the 10-week treatment period. These parameters included body weight, abdominal circumference, body length, glycemia, and blood pressure. Body mass index (BMI) was calculated by dividing the body weight (g) by the length (cm). The length of the rats was measured between the nasal and tail. Abdominal circumference (cm) was determined using a measuring tape around the anterior abdomen. For glycemia determination, blood was collected from the tail vein using the FreeStyle Precision Neo system (Abbott, Amadora, Portugal). Systolic blood pressure (SBP) and diastolic blood pressure (DBP) were measured using a noninvasive computerized tail-cuff IITC blood pressure system (CODA Non-Invasive Blood Pressure System, Kent Scientific Corporation, CT, USA). For this, rats were warmed for 30 min at 28 °C in a bag before each measurement to obtain a steady pulse level. Three readings were taken consecutively, and the average was then calculated. The duration of the experimental period (10 weeks) was established after confirmation that animals in the FRUCT group presented increased SBP, DPB, and glycemia.

Food and fluid intake were monitored every day for 10 weeks and energy ingestion was calculated (by multiplying food or fluid ingestion values by the corresponding reference energy values). The feed efficiency ratio was calculated as mean body weight gain (g)/food intake (g) × 100.

#### 2.3.4. Oral Glucose Tolerance Test (OGTT) and Insulin Tolerance Test (ITT)

After a 6 h fasting period, the oral glucose tolerance test (OGTT) was performed by administration of glucose (1 g/kg body weight, by gavage) to the rats. Glycemia was measured in blood collected from the tail vein (at 0, 15, 30, 60, 90, and 120 min after glucose administration) using a FreeStyle Precision Neo system (Abbott, Amadora, Portugal). The insulin tolerance test (ITT) was performed in 6 h fasted animals by administering insulin (0.75 U/kg body weight, i.p., Actrapid^®^, Novo Nordisk^®^, Paço de Arcos, Portugal), with blood glycemia measured as mentioned above. Both tests were performed at the 9th week of treatment.

#### 2.3.5. Collection of Blood and Tissue Samples After Euthanasia

At the end of the 10th week of treatment, and after a 6 h fasting period, the animals were euthanized with sodium phenobarbital (100 mg/kg of body weight, i.p., Euthanimal, Nephar). After induction of deep anesthesia, heparin (200 IU) (B. Braun, Amadora, Portugal) was injected through the right femoral vein. Blood was collected by cardiac puncture and plasma and serum were separated and stored at −80 °C. Liver and MAT were dissected, weighed and immediately frozen in liquid nitrogen and stored at −80 °C or fixed in 10% formaldehyde and processed to paraffin.

### 2.4. Determination of Plasma Biochemical Parameters

Plasma biochemical markers were measured in the Central Laboratory, Department of Clinical Pathology, Centro Hospitalar Universitário São João, using conventional methods with an AU5400 automated clinical chemistry analyzer (Beckman-Coutler, Paço de Arcos, Portugal). The measurements included hepatic function markers (aspartate aminotransferase, alanine aminotransferase, and alkaline phosphatase) and metabolic status markers (glucose, uric acid, triacylglycerides, total cholesterol, very low-density lipoprotein cholesterol, low-density lipoprotein cholesterol, and high-density lipoprotein cholesterol). C-reactive protein, total bilirubin, direct bilirubin, sodium, potassium, chloride, magnesium, calcium, and phosphorus were also determined.

### 2.5. Quantification of Plasma Insulin, Leptin, and Angiotensin II Levels

Fasting insulin levels were measured using a rat insulin enzyme-linked immunosorbent assay (ELISA) Kit (10-1250-01, Mercodia, Uppsala, Sweden). The homeostasis model assessment for insulin resistance (HOMA-IR) and for β-cell function (HOMA-β) were determined using the following Equations (1) and (2), respectively:

HOMA-IR = [FI × FG/22.5](1)

HOMA-β = [(20 × FI)/(FG − 3.5)](2)

where FI corresponds to fasting insulin levels (mU/mL) and FG to fasting glucose levels (mmol/L); 22.5, 20, and 3.5 are scaling factors used to normalize the results of the HOMA formula.

Plasma leptin levels were measured using a rat leptin ELISA kit (E-EL-R0582, Elabscience, Houston, TX, USA). Plasma angiotensin II levels were measured using a rat angiotensin II ELISA kit (E-EL-R1430, Elabscience, Houston, TX, USA). All the assays were performed according to the manufacturer’s instructions. 

### 2.6. Evaluation of Systemic Inflammatory Markers

#### 2.6.1. N-Acetylglucosaminidase (NAG) Serum Levels

Systemic inflammation was assessed by measuring the serum levels of α-N-acetylglucosaminidase (NAG), an enzyme highly expressed in activated macrophages. For this, after incubation of the serum (100 μL) for 10 min at 37 °C with 100 μL of p-nitrophenyl-N-acetyl-β-D-glucosaminide (2.24 mM) (Sigma-Aldrich, St. Louis, MO, USA) prepared in citrate/phosphate buffer (0.1 M citric acid, 0.1 M Na_2_HPO_4_; pH 4.5), the reaction was stopped by the addition of 100 μL of 0.2 M glycine buffer (pH 10.6). Substrate hydrolysis was measured at 405 nm in a microplate reader (Thermo Fisher Scientific, Waltham, MA, USA). Results were expressed as NAG concentration (mmol/mL).

#### 2.6.2. Nitric Oxide (NO) Serum Levels

Nitric oxide (NO) production was measured using the Griess reagent method [35]. In brief, after incubation of the serum (100 μL) with an equal volume of Griess reagent for 15 min (at room temperature), absorbance was measured at 550 nm in a microplate reader (Thermo Fisher Scientific, Waltham, MA, USA). Results were expressed as NO concentration (μM).

### 2.7. Histological Analysis

#### 2.7.1. Evaluation of Hepatic Fibrosis, Steatosis, Inflammation, and Ballooning

Liver tissue was fixed in 10% formaldehyde for 48 h at 4 °C, dehydrated, and embedded in paraffin. Subsequently, 3 and 12 μm thick sections were obtained with a Leica^®^ Microtome (Leica^®^ RM2125RT, Vila Nova de Famalicão, Portugal) and stained according to the following protocols.

Liver fibrosis was evaluated in 3 μm liver sections stained with Sirius Red (Sigma-Aldrich, St. Louis, MO, USA). Briefly, the slides were incubated with 0.5% of Sirius Red solution dissolved in saturated picric acid for 90 min, then washed in acidified water and finally mounted with Entellan. For each animal, five randomly stained sections were photographed at 200× magnification (Nikon ECLIPSE 50i, Tokyo, Japan) and analyzed using an ImageJ software^®^ version 2.14.0 device (National Institutes of Health, Bethesda, MD, USA). The area of positive Sirius red staining was calculated as the area of positive staining per total area, excluding areas with blood vessels.

Histological alterations were evaluated in 3 μm liver sections stained with Hematoxylin and Eosin (H&E) (Sigma-Aldrich, St. Louis, MO, USA). All slides for each animal were then examined and scored for the severity of liver steatosis, lobular inflammation, and ballooning by an experienced pathologist (J.L.) in a blinded manner under a compound light microscope at 200× magnification (Nikon ECLIPSE 50i, London, UK). The metabolic dysfunction-associated steatohepatitis (MASH) histological scoring system for metabolic dysfunction-associated steatotic liver disease (MASLD activity score) was used to assess the extent of steatosis with grade S_0_–S_3_ (S_0_: < 5%; S_1_: 5–33%; S_2_: 34–66%; S3: > 66%), to assess hepatocyte ballooning with grade 0–2 (0, none; 1, clusters of hepatocytes with rounded shape and pale and/or reticulated cytoplasm; 2, same as score 1 with enlarged hepatocytes (>2×normal size)) and to assess the extent of lobular inflammation with grade 0–2 (0, none; 1, ≤2 foci per 20× field; 2, >2 foci per 20× field) [36].

#### 2.7.2. Determination of Hepatic Lipid Accumulation

Hepatic lipid accumulation was assessed in 12 μm liver cryo-sections stained with Oil Red O (ORO) (Sigma-Aldrich, St. Louis, MO, USA), briefly counterstained with hematoxylin, according to an optimized protocol [37]. For each animal, ten randomly stained sections were photographed at 200× magnification (Nikon ECLIPSE 50i, UK) and analyzed using an ImageJ software^®^ version 2.14.0 device (National Institutes of Health, Bethesda, MD, USA). The ORO staining area was calculated as the area of positive staining per total area.

#### 2.7.3. Quantification of Hepatic Glycogen Content

Glycogen content was quantified in 3 μm liver sections stained with Periodic Acid-Schiff (PAS) (Sigma-Aldrich, St. Louis, MO, USA). Briefly, slides were oxidized in periodic acid (10%) for 10 min, rinsed in running water for 5 min, and stained with Schiff’s solution for 30 min. Counterstaining was performed with hematoxylin for 10 s. For each animal, five randomly stained sections were photographed at 200× magnification (Nikon ECLIPSE 50i, UK) and analyzed using an ImageJ software^®^ version 2.14.0 device (National Institutes of Health, Bethesda, MD, USA). The PAS staining area was calculated as the area of positive staining per total area.

#### 2.7.4. Evaluation of Mesenteric Adipose Tissue (MAT) Hypertrophy

MAT was fixed in 10% formaldehyde for 48 h at 4 °C and then dehydrated and embedded in paraffin. A Leica^®^ microtome (Leica^®^ RM2125RT, Vila Nova de Famalicão, Portugal) was used to obtain 3 μm thick sections, which were then stained with H&E (Sigma-Aldrich, St. Louis, MO, USA). Four random images from each tissue section were captured at 200× magnification for each animal using a Zeiss Axioskop 40 light microscope and Leica EC3 digital camera (Nikon ECLIPSE 50i, UK) ImageJ software^®^ version 2.14.0 device (National Institutes of Health, Bethesda, MD, USA) was used to analyze the area of adipocytes.

### 2.8. Quantitative Real-Time Polymerase Chain Reaction (RT-PCR)

Total ribonucleic acid (RNA) was isolated from frozen liver and MAT samples using NZYol (NZYtech, Lisbon, Portugal) and the concentrations were determined by a NanoDrop One (Thermo Fisher Scientific, Waltham, MA, USA). Total RNA was synthesized using a qScript cDNA SuperMix (Quanta Biosciences, Beverly, MA, USA), following the manufacturer’s instructions. Quantitative real-time polymerase chain reaction (RT-qPCR) was then performed on a LightCycler 96 System (Roche, Mannheim, Germany) utilizing the KAPA SYBR FAST qPCR master mix (2x) kit (Kapa Biosystems, Wilmington, NC, USA), following the manufacturer’s instructions. The sequences and cycling conditions of primers are listed in Supplementary Table S2. The 2^−ΔΔCT^ method [38] was used to determine the relative expression of the target genes normalized to glyceraldehyde 3-phosphate dehydrogenase (GAPDH).

### 2.9. Determination of Hepatic Oxidative Stress Markers

#### 2.9.1. Quantification of Thiobarbituric Acid Reactive Substance (TBARS) Levels

Levels of thiobarbituric acid reactive substances (TBARSs), as a marker of lipid peroxidation, were measured in liver homogenates using a lipid peroxidation (MDA) assay kit (MAK085, Sigma-Aldrich, St. Louis, MO, USA). Results were expressed as malondialdehyde (MDA) concentration (nmol/mg).

#### 2.9.2. Quantification of Glutathione (GSH) Levels

Total glutathione (total GSH) and oxidized glutathione (GSSG) content in liver homogenates were measured using a glutathione colorimetric detection kit (EIAGSHC, Thermo Fisher Scientific, Waltham, MA, USA) following the manufacturer’s instructions. Reduced glutathione levels (red GSH) were determined using Equation (3):

Red GSH = Total GSH − GSSG(3)

The data were expressed as µM of GSH.

#### 2.9.3. Determination of Xanthine Oxidase (XO) Activity

Xanthine oxidase (XO) activity was assayed by measuring the conversion of xanthine to uric acid following the xanthine oxidase activity Assay Kit (MAK078, Sigma-Aldrich, St. Louis, MO, USA) according to the manufacturer’s instructions. The results were expressed as XO activity (mmol/min/mg).

### 2.10. Statistical Analysis

Data were expressed as arithmetic mean ± standard error of the mean (S.E.M.). n indicates the number of animals. Statistical significance of the difference between two groups was assessed by Student’s *t*-test, and statistical significance of the difference between more than two groups was assessed by one-way ANOVA followed by Turkey’s post-hoc multiple comparison test. Analyses were performed using GraphPad Prism version 9.0 software (San Diego, CA, USA). Differences were considered statistically significant at *p* < 0.05.

## 3. Results

### 3.1. The Dried Coffee Pulp Contains a High Content of Caffeine, Chlorogenic Acids, and Fiber

Supplementary Table S1 shows the chemical composition of the dried CP used in this study. The dried CP exhibited a high content of total minerals (5.58 g/100 g), crude proteins (6.16 g/100 g), and total carbohydrates (75.42 g/100 g), of which 33.86 g/100 g corresponded to dietary fiber, mainly insoluble ones (27.87 g/100 g). The CP also displayed a low-fat content, mainly composed of unsaturated fatty acids (54.7%)—oleic, linoleic, and linolenic acids—alongside a substantial proportion of the saturated palmitic acid (36.95%).

Compared with the standard feed diet (Supplementary Table S1), the dried CP contained lower levels of protein and fat but significantly higher amounts of dietary fiber, both soluble and insoluble. Furthermore, incorporating dried CP into the animal’s diet led to the consumption of phytochemicals absent in the standard feed, such caffeine and phenolic compounds (chlorogenic acids), which were present in the CP in considerable quantities.

### 3.2. Coffee Pulp Reduced Weight Gain, Ameliorated the Increase in Blood Pressure, Improved Blood Glucose Levels, and Reduced Insulin Resistance in Fructose-Fed Rats

**Figure 2:**
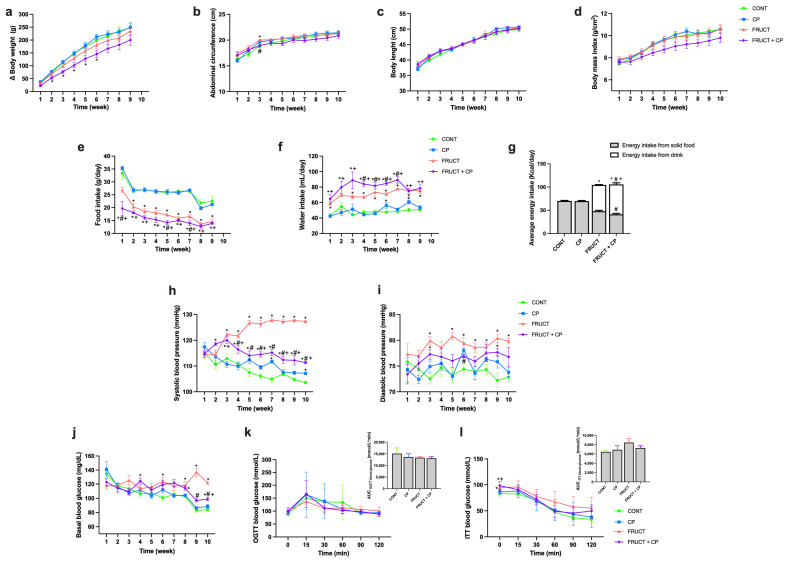
Coffee pulp reduced weight gain, ameliorated the increase in blood pressure, improved blood glucose levels, and reduced insulin resistance in fructose-fed rats. (**a**) Δ body weight, (**b**) abdominal circumference, (**c**) body length, (**d**) body mass index (BMI), (**e**) food intake, (**f**) water intake, (**g**) average caloric intake originating from solid food or drink, (**h**) systolic blood pressure (SBP), (**i**) diastolic blood pressure (DBP), (**j**) basal blood glucose, (**k**) oral glucose tolerance test (OGTT) and the respective area under the curve (AUC), and (**l**) insulin tolerance test and the respective AUC. Data are presented as means ± S.E.M; *n* = 6 per group. * *p* < 0.05 significantly different from CONT; ^#^ *p* < 0.05 significantly different from FRUCT; ^+^ *p* < 0.01 significantly different from CP (by one-way ANOVA followed by Tukey’s post-hoc test).

As shown in Figure 2a, the animals from the FRUCT + CP group had a significantly lower body weight than the CONT group starting from the second week until the seventh week, and this tendency continued to be observed until the end of the experiment. No major differences in abdominal circumference and body length were observed (Figure 2b,c), although a tendency for a lower BMI in the FRUCT + CP animals was observed (Figure 2d).

Regarding food and fluid consumption, the animals in the FRUCT and FRUCT + CP groups had a significantly lower daily food intake and a significantly higher volume of fluid consumption than those in the CONT and CP groups (Figure 2e,f). These differences were observed throughout the entire period of the dietary intervention. Alone, CP produced no change in fluid and food consumption. However, when combined with fructose (FRUCT + CP group), a lower daily food intake and a higher fluid intake were observed when compared to the FRUCT group, during some weeks of the intervention period (Figure 2e,f). Importantly, the average daily energy intake in fructose-drinking rats (FRUCT and FRUCT + CP groups) was significantly higher than in rats receiving water (CONT and CP groups), with approximately 57% and 65%, respectively, of this energy coming from the fructose-containing drink. It should also be emphasized that the average daily energy intake from solid food was significantly lower and the average daily energy intake from drink was significantly higher in the FRUCT + CP animals than in the FRUCT animals (Figure 2g).

In relation to the blood pressure, the SBP and DBP in the FRUCT group were significantly higher than in the CONT group from the third week onwards. Importantly, from the fourth week until the end of the dietary intervention, the FRUCT + CP animals had a significantly lower SBP compared to the FRUCT animals, and a similar trend in the DBP levels was found (Figure 2h,i). So, CP appears to be able to attenuate the fructose-induced increase in SBP. Notwithstanding, the changes found in SBP and DBP do not appear to be related to alterations in serum angiotensin II levels (Table 1).

Regarding glucose homeostasis, higher fasting glycemia was found in the FRUCT (in weeks 4, 8, and 10) and FRUCT + CP (in weeks 6, 8–10) groups. However, it is important to note that fasting glycemia in the FRUCT + CP group was considerably lower in the last two weeks of the intervention when compared to the FRUCT group (Figure 2j and Table 1). Furthermore, at the end of the experimental period, higher fasting plasma levels of insulin and a higher HOMA-IR index were found in the FRUCT group when compared to the CONT group. Interestingly, the combined consumption of fructose and CP (FRUCT + CP group) led to a decrease in plasma insulin concentrations and in the HOMA-IR index to values similar to those of the CONT group (Table 1). Despite these differences observed at the end of the experimental period, no differences in glucose tolerance or insulin resistance were noted, as assessed by OGTT and ITT at week 9 of the treatment (Figure 2k,l). Overall, these results suggest that CP interferes with glucose homeostasis, being able to reverse the effects of excessive fructose consumption on fasting glycemia and insulinemia.

In addition, the consumption of fructose (FRUCT group) led to an increase in plasma leptin and TAG levels, which were not observed in the FRUCT + CP group (Table 1). At the inflammatory level, although no differences in CRP and NO levels were observed, NAG levels were raised in the FRUCT group when compared to the CONT animals, and this difference disappeared in the FRUCT + CP group. Lastly, no differences in plasma electrolyte content were found (Table 1).

This set of findings suggests that the long-term ingestion of CP is able to revert some of the metabolic dysregulation induced by fructose drinking, namely the increase in body weight, blood pressure, and fasting glycemia, and insulin resistance.

### 3.3. Coffee Pulp Improves Plasma Biomarkers of Liver Injury and Reverses Hepatic Lipid Accumulation in Rats Fed a Fructose-Rich Diet

The liver is a key organ for maintaining normal glucose homeostasis through a balance between glucose production and glucose storage [39]. However, this balance is compromised in insulin-resistant states, which are often seen with high intakes of fructose-containing beverages or foods [40]. In order to assess the capacity of CP to attenuate liver damage in the presence of a high-fructose diet, we evaluated plasma biomarkers and liver morphological and histopathological features known to be associated with liver injury.

We did not observe significant variations in key indicators of liver damage (plasma levels of AST, ALT, ALP, ALB, BILT, and UA), between groups. Nevertheless, plasma BILD levels were significantly lower in the CP and FRUCT + CP groups as compared to the CONT group (Table 1), suggesting that CP may help to improve liver function.

**Figure 3:**
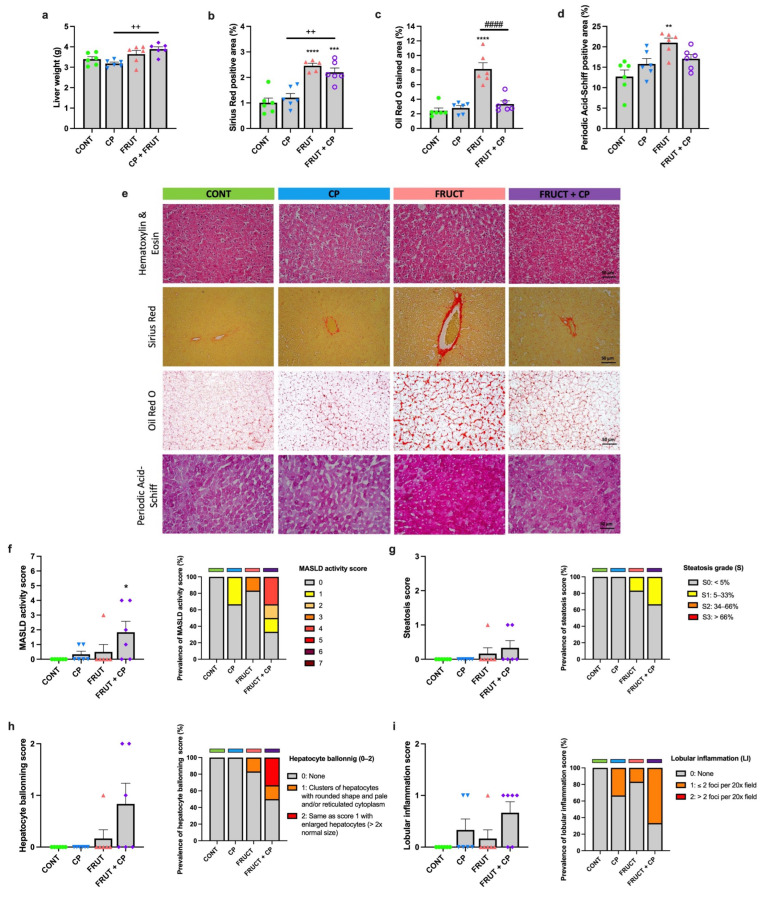
Coffee pulp reverses hepatic lipid and glycogen accumulation in rats fed a fructose-rich diet. (**a**) Liver weight, (**b**) area of Sirius Red-stained liver section, (**c**) area of Oil-Red-O-stained liver section, (**d**) area of Periodic Acid-Schiff-stained liver section, (**e**) representative images of liver sections stained with Sirius Red, Oil Red O, and Periodic Acid-Schiff (200× magnification; scale bar: 50 µm), (**f**) MASLD activity score, (**g**) steatosis score, (**h**) hepatocyte ballooning score, and (**i**) lobular inflammation score. Data are presented as means ± S.E.M, *n* = 6 per group. * *p* < 0.05, ** *p* < 0.01 *** *p* < 0.001 **** *p* < 0.0001 significantly different from CONT; ^++^ *p* < 0.01 significantly different from CP; ^####^ *p* < 0.0001 significantly different from FRUCT (by one-way ANOVA followed by Tukey’s post-hoc test).

Regarding liver weights, it was higher in the FRUCT + CP group than in the CP group (Figure 3a). The evaluation of hepatic fibrosis, as assessed by Sirius Red staining, shows that CP was not able to reduce the fructose-induced hepatic fibrosis (Figure 3b,e). In contrast, when combined with fructose, CP effectively abolished the increase in both hepatic fat accumulation (Figure 3c,e) and glycogen storage (Figure 3d,e) induced by fructose (FRUCT rats).

Dysregulation in glycogen metabolism and ectopic fat accumulation in parenchymatous organs, such as the liver, can drive inflammation, lipid accumulation, and fibrosis, resulting in MASLD and its progressive form, MASH [41]. So, we decided to perform liver histological analyses to determine the impact of CP on MASLD prevention. So, based on the MASH score, liver steatosis, hepatocyte ballooning, and lobular inflammation were evaluated (Figure 3f–i). As shown in Figure 3f, animals of the FRUT + CP group have a significantly higher MASLD activity score (Figure 3g), although no differences were observed in hepatic steatosis, hepatocyte ballooning, and lobular inflammation grades (Figure 3g–i).

### 3.4. Coffee Pulp Interferes with the Changes in the mRNA Expression of Key Genes Involved in Hepatic Glucose and Lipid Metabolism in Rats Fed a Fructose-Rich Diet

**Figure 4:**
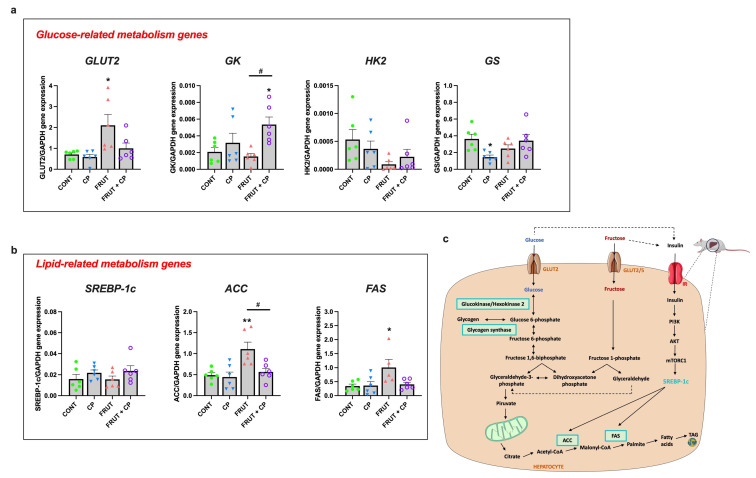
Coffee pulp interferes with the changes in the expression of key genes involved in hepatic glucose and lipid metabolism in rats fed a fructose-rich diet. (**a**) mRNA levels of glucose-related metabolism genes (glucose transporter 2 (*GLUT2*), glucokinase (*GK*), hexokinase II (*HK2*), and glycogen synthetase (*GS*), (**b**) mRNA levels of lipid-related metabolism genes (acyl-CoA carboxylase (*ACC*), fatty acid synthase (*FAS*), and sterol regulatory element-binding protein 1 (*SREBP-1c*), and (**c**) schematic depiction of representative genes and pathways involved in glucose and lipid metabolism in the liver. Data were normalized to the expression of glyceraldehyde-3-phosphate dehydrogenase (*GAPDH*). Data are presented as means ± S.E.M; *n* = 6 per group. * *p* < 0.05, ** *p* < 0.01 significantly different from CONT; ^#^ *p* < 0.05 significantly different from FRUCT (by one-way ANOVA followed by Tukey’s post-hoc test).

Next, given the importance of the liver in carbohydrate and lipid metabolism, we assessed the effect of fructose and/or CP on the mRNA expression of key genes involved in hepatic glucose metabolism. The mRNA levels of glucose transporter 2 (*GLUT2*), the glucose transporter responsible for glucose entry into hepatocytes, were significantly increased in FRUCT rats when compared to CONT rats, and this difference disappeared in the FRUCT + CP group. The mRNA levels of glucokinase (*GK*), the main hepatic enzyme responsible for the conversion of glucose into glucose-6-phosphate (G6P), were considerably increased in FRUCT + CP animals, both in relation to the CONT group and to the FRUCT group. Finally, the expression of glycogen synthetase (*GS*), a key enzyme in glycogenesis, was significantly reduced in the CP group, when compared to the CONT group. In contrast, no differences were found in the mRNA levels of hexokinase 2 (*HK2*), an enzyme that also phosphorylates glucose into G6P (Figure 4a).

In addition to glucose metabolism, the liver also plays a critical role in fat metabolism. Thus, we evaluated the effect of fructose and/or CP on the mRNA levels of some lipid metabolism-related genes. No differences were found in *SREBP-1c* (sterol regulatory element-binding protein 1) mRNA levels. However, the mRNA levels of *ACC* (acetyl-CoA carboxylase) and *FAS* (fatty acid synthase), two key enzymes involved in fatty acid synthesis, which were increased in FRUCT animals, returned to CONT levels in the FRUCT + CP group (Figure 4b).

### 3.5. Coffee Pulp Abrogates the Effects of a Fructose-Rich Diet on Hepatic Lipid Peroxidation and Glutathione Levels

A high-fructose diet can increase fat accumulation in the liver, and consequently, increase reactive oxygen species (ROS) and lipid peroxidation levels, leading to liver cell damage [40]. Oxidative stress was, therefore, assessed in liver samples from all groups of animals by the quantification of glutathione and MDA levels and by the determination of XO activity.

**Figure 5:**
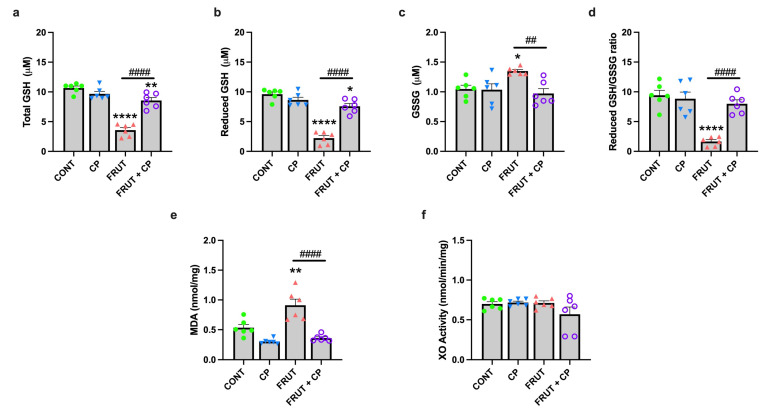
Coffee pulp abrogates the effects of a fructose-rich diet on hepatic lipid peroxidation and glutathione levels. (**a**) Total glutathione (total GSH), (**b**) reduced glutathione (red GSH), (**c**) oxidized glutathione (GSSG), (**d**) reduced glutathione/oxidized glutathione ratio (red GSH/GSSG ratio), (**e**) malondialdehyde (MDA) levels, and (**f**) xanthine oxidase (XO) activity. Data are presented as means ± S.E.M; *n* = 6 per group. * *p* < 0.05, ** *p* < 0.01, **** *p* < 0.0001 significantly different from CONT; ^##^ *p* < 0.01, ^####^ *p* < 0.0001, significantly different from FRUCT (by one-way ANOVA followed by Tukey’s post-hoc test).

Glutathione (GSH) is the main intracellular antioxidant buffer against oxidative stress and exists mainly in the form of reduced GSH and GSSG. Our data revealed that the FRUCT group exhibited a significant reduction in hepatic total and reduced GSH, associated with an increase in GSSG levels, resulting in a decrease in reduced GSH/GSSG ratio. Interestingly, all these differences were abolished in the FRUT + CP animals (Figure 5a–d).

MDA is an end product of lipid peroxidation and has been used as a biomarker of oxidative stress. As expected, hepatic MDA levels in the animals of the FRUCT group were significantly higher when compared to CONT animals. However, the combined consumption of fructose and CP (FRUCT + CP group) was able to suppress this difference (Figure 5e). Finally, no differences were found in the hepatic XO activity (an enzyme that catalyzes the oxidation of hypoxanthine to xanthine, and of xanthine to UA along with the generation of ROS) (Figure 5f).

Collectively, these results indicate that CP was able to completely abolish the effects induced by a high fructose diet on hepatic MDA, total GSH, red GSH, GSSG levels, and the red GSH/GSSG ratio, suggesting that CP improves hepatic antioxidant capacity.

### 3.6. Coffee Pulp Interferes with the Changes in Key Genes Involved in Mesenteric Adipose Tissue Glucose Metabolism in Rats Fed a Fructose-Rich Diet

**Figure 6:**
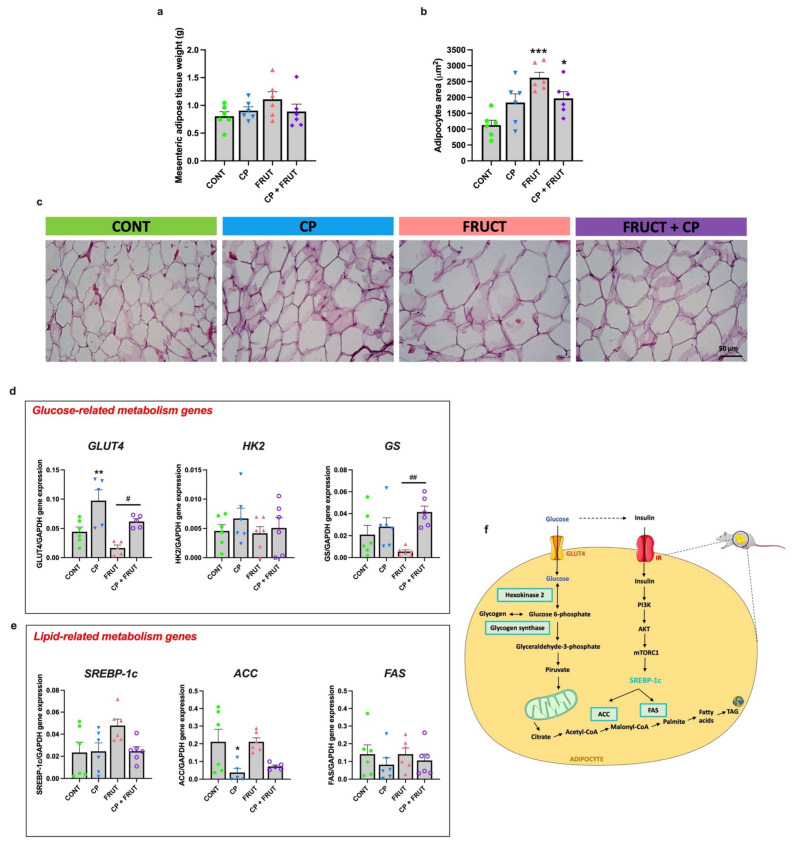
Coffee pulp interferes with the changes in the expression of key genes involved in mesenteric adipose tissue (MAT) glucose metabolism in rats fed a fructose-rich diet. (**a**) MAT weight, (**b**) average cross-sectional area of the MAT adipocytes, and (**c**) representative images of hematoxylin-eosin-stained MAT sections (200× magnification; scale bar: 50 µm), (**d**) mRNA levels of glucose-related metabolism genes (glucose transporter 4 (*GLUT4*), hexokinase II (*HK2*), and glycogen synthetase (*GS*) and (**e**) mRNA levels of lipid-related metabolism genes (acyl-CoA carboxylase (*ACC*), fatty acid synthase (*FAS*), and sterol regulatory element-binding protein 1 (*SREBP-1c*), (**f**) schematic depiction of representative genes and pathways involved in glucose and lipid metabolism in adipose tissue. Data were normalized to the expression of glyceraldehyde-3-phosphate dehydrogenase (*GAPDH*). Data are presented as means ± S.E.M; *n* = 6 per group. * *p* < 0.05, ** *p* < 0.01, *** *p* < 0.001 significantly different from CONT; ^#^ *p* < 0.05, ^##^ *p* < 0.01 significantly different from FRUCT (by one-way ANOVA followed by Tukey’s post-hoc test).

AT is involved in the maintenance of energy homeostasis through the storage and release of lipids in response to systemic dietary and metabolic demands [42]. Fructose is known to increase AT lipid accumulation and to cause adipocyte hypertrophy [43]. In this sense, the ability of CP to attenuate MAT hypertrophy in the presence of a high fructose diet was evaluated. As shown in Figure 6a, the consumption of CP, fructose, or both did not affect MAT weight. However, as indicated by H&E staining, an increase in MAT adipocyte size in the FRUCT and FRUCT + CP groups, in relation to the CONT group, was found. Notwithstanding, the adipocyte area of the FRUCT + CP group tends to be smaller than that of the FRUCT group (Figure 6b,c).

Lastly, we explored the effects of fructose and/or CP on key genes involved in MAT glucose (glucose transporter 4 (GLUT4), HK2 and GS) and lipid (SREBP-1c, ACC and FAS) metabolism, by RT-qPCR. *GLUT4* mRNA levels were significantly increased in CP animals when compared to CONT animals and in the FRUCT + CP animals in relation to FRUCT animals. In contrast, no difference between groups was observed in the expression of *HK2*. In relation to GS, its mRNA levels were significantly higher in the FRUCT + CP group, compared to the FRUCT group (Figure 6d). Finally, no differences in lipid metabolism-related gene expression were found, apart from a reduction in *ACC* mRNA levels in the CP group, when compared to the CONT group (Figure 6e).

Taken together, these results indicate that although CP is not able to reverse fructose-induced MAT hypertrophy; it reduces and/or abolishes the effect of fructose-feeding on the mRNA expression of key genes involved in adipose tissue glucose (GLUT4 and GS) metabolism.

## 4. Discussion

MetS can be caused by dietary changes, such as a diet high in fat and refined carbohydrates, such as fructose [44]. Induction of MetS in a high-fructose diet animal model is considered a valid experimental strategy because it recapitulates the metabolic changes observed in humans, including insulin resistance, hyperglycemia, hypertension, dyslipidemia, and obesity [44,45]. Considering these factors, we used a high-fructose diet to induce MetS in order to evaluate the effect of CP on metabolic and physiological dysregulations associated with MetS. The preventive effect of CP consumption on body weight gain, hyperglycemia, hypertension, insulin resistance, and oxidative stress has been reported in in vitro [21,23,24,25] and in vivo [26,28] studies. However, studies demonstrating the in vivo metabolic benefits of CP remain scarce, and used other experimental models for MetS induction, namely the high-fat diet. This study, therefore, represents a significant step forward in understanding the benefits of CP in relation to MetS associated with a high fructose diet.

The primary bioactive components identified in CP are caffeine, chlorogenic acids, and fiber [13,14,16]. Our analysis revealed that our sample of CP is particularly rich in fiber, with a high content of insoluble dietary fiber, caffeine, and chlorogenic acids, specifically 5-caffeoylquinic acid.

In the present study, the consumption of FRUCT + CP significantly reduced body weight gain compared to the CONT rats until the seventh week of the experimental period, and this tendency (and also in abdominal circumference and BMI) continued until the end of the treatment. Consistent with our findings, a previous study reported a reduction in body weight in high-fat-diet-fed rats supplemented with CP [28]. Of note, a clear shift in food intake (decrease) and fluid intake (increase) in fructose-fed rats (FRUCT and FRUCT + CP groups) was observed, pointing to a sweet taste preference of the animals. Importantly, the reduction in body weight increase in FRUCT + CP animals happened despite the fact that the energy intake of these animals was higher than that of CONT animals, and this reduction in weight gain was not observed in FRUCT animals, which had a total energy intake similar to that of FRUCT + CP animals (although differences in the fraction energy intake derived from solid and liquid sources in these two groups were found). These observations suggest that, when associated to a high-fructose diet, CP may alter energy expenditure and may also affect food preference, namely by increasing the sweet taste preference. One of the major components of CP, and particularly of our CP sample, is dietary fiber [8], and soluble dietary fiber has been reported to reduce obesity-related health problems by reducing food intake, weight gain, and adiposity [46]. Additionally, phenolic compounds, such as caffeine, are also found in high concentrations in CP, including our sample [8], and caffeine has also been reported to prevent body weight increase by stimulating thermogenesis, lipolysis, energy expenditure, and resting metabolic rate [47]. Based on the considerations outlined above, it is likely that both fiber and caffeine play an important role in the weight-reducing effects associated with CP.

Hypertension is a key component of the MetS and is closely associated to excessive fructose intake [48,49]. In the present study, the significant increase in SBP and DBP in the FRUCT group compared with the CONT group from the third week is consistent with the existing literature linking high-fructose intake to hypertension [45,48,49]. The mechanisms behind fructose-induced hypertension are complex and can involve (a) sympathetic nervous system overactivation, (b) increased renal and intestinal salt absorption, and (c) impaired endothelial function, associated with increased angiotensin II levels [48,49]. The lack of correlation, in our study, between changes in SBP/DBP and angiotensin II serum levels points to the conclusion that the fructose-induced hypertension was not mediated by an increase in angiotensin II levels, which is one of the mechanisms considered to be involved in fructose-induced hypertension [48].

Interestingly, CP was able to significantly reduce SBP in fructose-fed rats from the fourth week until the end of the dietary intervention, suggesting a potential protective role of CP in relation to fructose-induced hypertension. These findings are consistent with a previous study where CP normalized the SBP increased in rats fed a high-fat and high-carbohydrate diet [28]. Previous studies have also shown that certain dietary compounds present in high amounts in our CP sample, namely caffeine and chlorogenic acids, may attenuate the effects of a hypertension-inducing diet [50,51]. Caffeine can lower blood pressure by antagonizing adenosine receptors, such as A1R, which influences total peripheral resistance, diuresis, and heart rate or by improving vascular endothelial function, leading to vasodilation [52]. As to chlorogenic acid, it can contribute to blood pressure reduction by inhibiting NADPH oxidase, leading to a decrease in free radical production by the direct scavenging of free radicals, the stimulation of NO production via the endothelium-dependent pathway, or the inhibition of plasma angiotensin-converting enzyme [52,53]. It is important to note, however, that despite the reduction in SBP in FRUCT + CP rats in relation to the FRUCT rats, SBP levels remained higher than those in the CONT and CP groups throughout the study. This suggests that although CP may have some anti-hypertensive properties, the persistent effects of prolonged fructose consumption are substantial, and the complete attenuation of these effects may require more prolonged exposure to CP. So, while the findings suggest that CP may help to attenuate the adverse effects of fructose on blood pressure, further research is needed to clarify the mechanisms involved.

In this study, the increase in fasting plasma glucose and insulin levels and in the HOMA-IR index in rats fed the high fructose diet compared to control rats indicates that ten weeks of treatment with 20% fructose is able to disrupt glucose homeostasis and establish a state of insulin resistance, without affecting β-cell function (HOMA-β index). Although no differences were found in OGTT and ITT, a strong correlation between HOMA-IR and the insulin tolerance test in Wistar rats was found, suggesting that HOMA-IR is an effective marker for assessing insulin resistance in this animal model [54]. Interestingly, the consumption of CP for ten weeks in combination with a high-fructose diet reduced fasting plasma glucose and insulin levels as well as the HOMA-IR index compared to the FRUCT rats. This indicates that CP, by simultaneously improving glucose tolerance and insulin sensitivity, was able to counteract the adverse consequences of excessive fructose intake on glucose homeostasis. The impact of coffee manufacturing by-products, such as CP, on glucose homeostasis remains largely unexplored, and our work is one of the first to address this issue.

This study was based on the concept that CP is a rich source of some of the same bioactive compounds found in coffee, albeit in different proportions. Coffee consumption is associated with significant improvement in glucose homeostasis in MetS [55,56], and caffeine is thought to be a major contributor to this effect of coffee by causing an increase in insulin secretion from pancreatic β-cells [57], glucose tolerance [58], and insulin sensitivity [58,59]. Additionally, chlorogenic acid has also been associated with a protective antihyperglycemic effect on T2D through several mechanisms: by enhancing insulin sensitivity [60], inhibiting G6P displacement enzymes [61], and decreasing glucose absorption [58]. In addition, its antioxidant properties appear to help reduce the oxidative stress associated with insulin resistance and T2D [47].

Leptin, mainly produced by the AT, participates in the regulation of food intake, body weight, energy expenditure, and adiposity, and its blood levels are usually higher in obese and insulin resistant individuals [62]. The leptin resistance observed in obesity also contributes to the onset of T2D [63]. In this study, and as expected, hyperleptinemia was observed in rats fed high fructose. The plasma leptin levels in FRUCT animals seem to be directly correlated with elevated plasma insulin concentration and insulin resistance observed in this group of animals. Although the high leptin levels found in the fructose-fed rats predicted a reduction in food intake and body fat; an increase in energy intake with no change in body weight was observed in the FRUCT animals. Importantly, in the FRUCT + CP group of animals, the hyperleptinemia observed in the FRUCT animals was no longer observed. Interestingly, this effect is similar to the effect of CP on insulin levels and insulin resistance in fructose-fed rats, in agreement with the literature that show a correlation between these parameters [64]. Only a few studies have investigated the effect of coffee on leptin levels, resulting in inconsistent results. Similar to our results, an observational study found no significant correlation between leptin levels and coffee consumption [65]. Conversely, other studies have shown that coffee components (i.e., caffeine) can reduce serum leptin in rats [66]. Further research is, therefore, needed about this topic.

As far as the lipid profile is concerned, plasma TAG levels were increased in FRUCT animals, compared to healthy animals, while no differences in plasma TC, HDL, VLDL, and LDL levels were seen. This result is consistent with the literature, as fructose feeding in rodents is associated with increased hepatic de novo lipogenesis, leading to increased TAG synthesis, and excessive fructose consumption is known to induce lipid profile disturbances [45,67]. Interestingly, CP supplementation in the fructose-fed rats ameliorated the increase in plasma TAG levels induced by fructose, suggesting that CP may play a protective role in lipid metabolism. Corroborating our results, Zhu et al. found that the long-term consumption of CP can reduce plasma TAG levels in mice fed a high-fat diet, thereby ameliorating obesity-related lipid accumulation [67].

At the inflammatory level, the FRUCT group exhibited higher NAG levels compared to the CONT animals, and this difference vanished in the FRUCT + CP group. Increased serum NAG levels may be associated with hepatic damage, including fibrosis [68], and our FRUCT group indeed showed signs of hepatic fibrosis, and will be discussed next.

MetS is a liver-centered disease, and MASLD constitutes the main cause of liver disease [69]. Histologically, MASLD is characterized by simple steatosis (lipid accumulation in the hepatocytes) without significant lobular inflammation or liver fibrosis. If left untreated, this condition may progress to MASH, which is characterized by balloniform degeneration of hepatocytes and diffuse lobular inflammation that may induce different stages of fibrosis. The progression towards fibrosis can lead to cirrhosis and even hepatocarcinoma [70]. Typically, fructose is recognized as one of the major mediators of MASLD, because a strong correlation exists between fructose intake and the degree of hepatic fibrosis, fat accumulation, inflammation, and oxidative stress [69]. So, we decided to investigate the potential MetS-improving properties of CP at the hepatic level in more detail.

The reduction in plasma BILD levels in both CP consumption groups (CP and FRUCT + CP groups) suggests a benefic effect of CP consumption on liver function. Indeed, elevated levels of BILD are often a sign of liver dysfunction, a key factor in the development of MetS [69].

From the histopathological analysis of hepatic tissue, we concluded that the high-fructose diet led to an increase in lipid accumulation, which interestingly was markedly diminished by CP consumption. Conversely, CP supplementation was not able to reverse the increase in hepatic fibrosis induced by fructose. Fibrosis in MASLD is frequently associated with an active necroinflammatory reaction (hepatic inflammation and hepatocyte ballooning) [71]. However, the increased fibrosis level in the FRUCT group was not associated with changes in MASLD score (which includes hepatocyte ballooning and lobular inflammation scores). Moreover, an increased MASLD score was observed in FRUCT + CP animals, although no significant differences in individual components (steatosis score, hepatocyte ballooning score, and LI score) were found. However, a tendency for higher hepatocyte ballooning score and LI scores in this group of animals appears to exist. So, the increase in MASLD score in FRUCT + CP livers is probably related to an increase in hepatocyte ballooning and inflammation. In summary, CP was able to reverse fructose-induced hepatic lipid accumulation but not fibrosis, and even tended to increase hepatocyte ballooning and hepatic inflammation. Importantly, portal fibrosis, which was actually the most prevalent pattern noted in the liver of the animals in our study, develops as MASLD worsens, indicating a later stage of liver damage [71]. Because our results showed that CP supplementation in FRUCT rats was not effective in reversing already established fibrosis, they suggest that CP could be more effective in less advanced MASLD. Because this is the first report of the effects of CP, in the context of a high fructose-feeding, on liver steatosis, fibrosis, and MAFLD score, it would be important to further clarify this point.

In order to identify the potential mechanism by which CP consumption reduced hepatic lipid accumulation in high-fructose fed rats, we investigate key lipid-related metabolism genes. In the FRUCT animals, a significant increase in hepatic *ACC* and *FAS* gene expression was found. ACC and FAS are crucial enzymes involved in de novo lipogenesis, being recognized as markers of adipogenesis [69], and their overexpression leads to excessive fatty acid production and hepatomegaly [72]. So, the fructose-feeding associated increase in hepatic lipid content appears to result from the activation of the de novo lipogenic pathway, which may lead to MASLD, if excessive. Both ACC and FAS are regulated by SREBP-1c [72]. Curiously, despite the significant upregulation of ACC and FAS gene expression, we did not find significant changes in *SREBP-1c* mRNA levels. Furthermore, liver weights remained comparable among the groups. The lower hepatic *ACC* and *FAS* expression in FRUCT + CP animals, compared to FRUCT animals, indicates that CP is able to mitigate the increase in de novo lipogenesis induced by fructose-feeding. In contrast to our observations, a previous study in mice on a high-fat diet showed that CP consumption resulted in a decrease in *SREBP-1c* and *FAS* mRNA levels, while *ACC* levels remained unchanged [67]. On the other hand, hepatic *SREBP-1c* and *ACC* mRNA levels were found to be lower in coffee polyphenols-fed mice than in high-fat control mice [73]. These discrepancies highlight the complexity of lipid metabolism regulation.

With regard to hepatic glycogen storage, we observed a higher glycogen content in the FRUCT rats compared to the CONT rats. This observation agrees with previous studies that have associated high fructose consumption with increased hepatic glycogen deposition [74]. Interestingly, our results showed that the addition of CP decreased the increased glycogen content seen in FRUCT rats, suggesting a role for CP in glycogen homeostasis. Further supporting the idea that CP may affect glycogen metabolism, a significant decrease in *GS* mRNA levels in CP rats compared with CONT rats was observed, suggesting that a decrease in GS activity after CP consumption reduces the conversion of glucose to glycogen, resulting in increased glucose oxidation. In hepatocytes, GLUT2 is responsible for controlling glucose uptake. Once inside the cell, glucose is phosphorylated to G6P by GK (mainly) or HK2 and subsequently metabolized by glycolysis or incorporated into glycogen [75]. Our study demonstrated a higher mRNA expression of *GLUT2* in FRUCT animals compared to CONT animals, possibly resulting in a higher hepatic capacity for glucose uptake. This adaptation may serve as a compensatory mechanism in response to the higher blood glucose levels of FRUCT animals [69,75]. Interestingly, our results also revealed a significant increase in *GK* mRNA expression in FRUT + CP animals compared to both CONT and FRUT rats. In the liver, GK acts as a sensor and helps modulate blood glucose levels by facilitating its uptake during hyperglycemia [76]. These results thus suggest that CP promotes the GK-mediated conversion of glucose to G6P, thereby offsetting the elevated glucose levels induced by fructose consumption and highlighting the potential role of CP in glucose homeostasis. Although HK2 also catalyzes the conversion of glucose to G6P, there were no significant differences in its mRNA levels between the groups. Nevertheless, although both HK2 and GK are able to phosphorylate glucose, GK is the predominant hexokinase expressed in the liver, accounting for approximately 95% of hexokinase activity in hepatocytes [76] and so the effect of CP on GK is more significant.

Oxidative stress is one of the causes of liver damage and high fructose intake is associated with an increase in hepatic oxidative stress, which contributes to insulin resistance, hepatic steatosis, MASLD, and MASH [40,41,77]. Remarkably, CP has emerged as a potential ally in mitigating these adverse effects. Indeed, CP shows promising antioxidant activity that may help protect the liver from oxidative stress [8,21,22,78]. Consistently, we observed a significant decrease in total GSH and reduced GSH levels and in the red GSH/GSSG ratio in the FRUCT group, together with a significant increase in GSSG levels. These results indicate a depletion of an important antioxidant defense in the liver and a progression to oxidative stress [79]. Of notice, a restoration of hepatic antioxidant status was observed in the FRUCT + CP group (improved levels of total GSH, reduced GSH, GSSG, and red GSH/GSSG ratio compared with FRUCT animals).

Also, the levels of MDA, a well-documented biomarker of lipid peroxidation, were significantly higher in the FRUCT group compared to the CONT group, confirming the premise that high fructose consumption exacerbates oxidative damage in the liver [40,77,80]. The reversal of this increase in the FRUCT + CP group to levels comparable to the CONT group reinforces the conclusion that CP can effectively mitigate hepatic oxidative damage, most probably because of its antioxidant components. Indeed, the phenolic and antioxidant content of CP appears to be responsible for the effective scavenging of free radicals and the enhancement of endogenous antioxidant systems [22]. In addition, studies have demonstrated that coffee consumption can lower hepatic MDA levels in rats fed a high-fructose diet [81].

On the other hand, the lack of substantial differences in hepatic XO activity between the groups suggests that changes in oxidative stress markers are likely to be due to changes in antioxidant availability and metabolism rather than direct changes in ROS production from XO-mediated purine metabolism [82]. Curiously, the observation that no significant differences in plasma UA levels exist between the groups further supports this conclusion. Overall, our findings indicate that the restoration of hepatic antioxidant status is particularly noteworthy, underscoring the potential role of CP in enhancing hepatic antioxidant capacity and reducing oxidative damage.

White adipose tissue (WAT) is an energy source that can adapt to fluctuations in the body’s energy needs and nutritional status [42,83]. WAT is essential for preserving lipid homeostasis, regulating body TAG levels, and plasma free fatty acids levels [83,84]. The dysfunction of WAT is a key factor in the development of insulin resistance and related disorders [83]. The liver is directly exposed to the cytokines and free fatty acids that are released from the MAT [85], suggesting that MAT may be more crucial in MetS-related diseases than other visceral adipose tissue stores [86]. With this in mind, we decided to also explore the potential benefit of CP on MAT metabolic health.

The fructose-fed animals (FRUCT and FRUCT + CP groups) exhibited similar MAT weights but had an enlarged MAT adipocyte size compared to the CONT and CP animals. So, CP treatment is not able to reverse the MAT hypertrophy induced by high-fructose intake. Notwithstanding, together with adipocyte enlargement in FRUCT animals, hyperleptinemia, also characteristic of MetS, was observed [1,2,11]. Although the FRUCT + CP group maintained an increased size of MAT adipocytes comparable to the FRUCT group, plasma leptin levels were similar to control levels, suggesting that a longer period of exposure to CP may be necessary to reduce MAT size. Hypertrophic MAT can result either from the formation of fewer but larger adipocytes or the accumulation of more lipids within existing adipocytes [83]. So, one would anticipate an increased expression level of genes associated with lipid synthesis in fructose-fed animals (FRUCT and FRUCT + CP) relative to the healthy groups (CONT and CP). However, contrary to these expectations, the expression of lipid metabolism-related genes (*ACC*, *FAS,* and *SREBP-1c*) remained comparable across all four groups, with the singular exception of the CP group, which showed a slight reduction in *ACC* mRNA levels compared to the CONT group. Differently from the present study, most studies have shown that coffee and its active compounds can improve adipose tissue lipid metabolism through the downregulation of lipogenic enzymes. For instance, Murase et al. showed that coffee polyphenols suppressed diet-induced body fat accumulation by downregulating *ACC*, *FAS,* and *SREBP-1c* in mice [73]. In another study, combination of caffeine and catechins synergistically inhibited lipid accumulation in 3T3-L1 adipocytes by regulating the expression of *FAS* [87]. In yet another study, the combination of chlorogenic acid and caffeine inhibited 3T3-L1 cell differentiation by downregulating *FAS* expression [88]. Of note, the alkaloid trigonelline, which is found in large quantities in CP, attenuated lipid accumulation by restricting adipocyte differentiation through the PPARγ cascade, together with the inhibition of adipocyte differentiation through the downregulation of *FAS* in 3T3-L1 cells [89]. It is important to emphasize that although our CP sample contains similar bioactive ingredients as those found in other coffee-derived compounds (e.g., caffeine and chlorogenic acids), the proportions of these individual components differ significantly from those found in other coffee extracts or even in different CP samples. These disparities in CP composition might be responsible for the discrepancies between our findings and those reported in previous studies. In addition, the methodologies used in our research are different; we used in vivo assays, whereas most other studies have focused primarily on in vitro experiments. This methodological divergence may also contribute to the different results observed in our study.

Concerning glucose metabolism, this study found that the expression of *GLUT4* was substantially increased in CP and FRUCT + CP groups when compared to the CONT and FRUCT groups, respectively. These findings underline the positive effects of CP in increasing *GLUT4* levels in MAT. GLUT4 is required for glucose uptake in insulin-sensitive tissues, such as the AT [77], and its reduction is normally associated with obesity-induced insulin resistance [90]. Thus, increased GLUT4 levels can improve glucose uptake and promote better glycemic control [77]. The increased *GLUT4* expression in MAT may play an important role in some of the beneficial effects of CP when associated with FRUCT, namely the reduction in plasma glucose and insulin levels, as well as the improvement in insulin resistance. A previous study with green coffee beans and rats fed a high-fat diet corroborated our results on adipose tissue GLUT4 levels [91].

In our study, MAT *HK2* expression did not show significant differences across the four groups. This may imply that while the glucose uptake mechanism via GLUT4 is upregulated in rats fed CP (CP and FRUCT + CP), the initial phosphorylation of glucose by HK2 remains unaffected, suggesting that other factors may modulate the subsequent metabolic pathways. Previous works have found a reduction in the AT expression of *HK2* in mice fed a high-fat diet, which consequently suppressed AT lipogenesis and promoted the release of nonesterified fatty acids [92]. Moreover, reduced *HK2* expression has also been observed in obese and diabetic patients, suggesting that the loss of *HK2* in AT may contribute to the development of insulin resistance and, thereby, hyperglycemia [92].

Although AT does not primarily store glycogen, it does express GS, which is dynamically regulated by several hormones, such as insulin [93]. Contrary to what was expected, our study showed a significant increase in *GS* expression in the FRUCT + CP group compared to the FRUCT group. This suggests that the MAT of FRUCT + CP animals may contain significantly higher levels of glycogen than CONT and FRUCT animals, highlighting an intriguing biological phenomenon that warrants further investigation. One potential mechanism posits that caffeine may modulate the gut microbiota [28], potentially leading to the production of short-chain fatty acids (SCFAs). SCFAs have been associated with improved metabolic profiles, including enhanced lipogenesis and glycogenesis in AT [94]. This shift in microbial metabolism could promote greater GS activity and glycogen storage within adipocytes. Further exploration of these mechanisms is essential to fully understand their implications.

## 5. Conclusions

Overall, the data presented in this study indicate that CP is able to effectively reverse several MetS-associated changes induced by a high-fructose diet in rats. The addition of CP to the diet of fructose-induced MetS rats not only prevented body weight gain, but also ameliorated hyperglycemia, insulin resistance, and hypertension. In addition, this coffee by-product was able to attenuate hepatic oxidative stress and ameliorate liver and AT dysfunction/lipotoxicity in this MetS model. We suggest that all these promising results may be related to the higher content of phenolic compounds, especially caffeine and chlorogenic acid (particularly, the 5-caffeoylquinic acid) present in CP.

Given these promising results, we can speculate that the regular consumption of CP may ameliorate and/or prevent some of the metabolic dysregulations associated with MetS in humans. In addition, adding value to this by-product will also contribute to the circular economy. However, future clinical trials are essential to validate our findings and explore the potential health benefits of CP in humans.

## Figures and Tables

**Table 1:** Effects of coffee pulp on plasma biochemical parameters (hepatic function markers, glucose homeostasis markers, hormone levels, lipid profile, inflammatory markers, and electrolytes).

Biochemical Parameters	CONT (*n* = 6)	CP (*n* = 6)	FRUCT (*n* = 6)	FRUCT + CP (*n* = 6)
**Hepatic function markers**				
Aspartate aminotransferase (AST) (U/L)	72.5 ± 6.3	67.7 ± 5.7	69.6 ± 11.1	69.8 ± 5.6
Alanine aminotransferase (ALT) (U/L)	25.0 ± 2.6	28.2 ± 4.5	25.8 ± 2.9	24.5 ± 1.9
Alkaline phosphatase (ALP) (U/L)	150.8 ± 25.0	142.7 ± 7.1	143.3 ± 17.0	188.5 ± 32.7
Albumin (ALB) (g/L)	25.3 ± 1.4	25.7 ± 0.7	27.1 ± 1.0	27.9 ± 1.6
Total bilirubin (BILT) (mg/dL)	0.16 ± 0.00	0.17 ± 0.01	0.13 ± 0.01	0.15 ± 0.01
Direct bilirubin (BILD) (mg/dL)	0.22 ± 0.07	0.06 ± 0.01 *	0.10 ± 0.02	0.05 ± 0.01 *
Uric acid (UA) (mg/dL)	1.6 ± 0.3	1.4 ± 0.2	2.2 ± 0.4	2.1 ± 0.4
**Glucose homeostasis**				
Glucose (GLU) (mg/dL)	197.3 ± 4.2	174.5 ± 5.9	264.8 ± 8.0 ****	212.3 ± 8.7 **###** ++
Insulin (INS) (μg/L)	1.41 ± 0.23	0.93 ± 0.03	2.79 ± 0.49 **	1.57 ± 0.19 **#**
HOMA-IR	17.36 ± 2.98	10.39 ± 0.56	46.15 ± 7.75 ***	20.49 ± 2.40 **##**
HOMA-β	94.09 ± 14.07	78.69 ± 3.53	124.51 ± 16.94	97.24 ± 14.72
**Hormone levels**				
Leptin (LEP) (ng/mL)	0.25 ± 0.04	0.24 ± 0.01	0.56 ± 0.09 **	0.45 ± 0.05 +
Angiotensin II (ANGII) (pg/mL)	48.48 ± 3.08	64.72 ± 3.43 *	63.14 ± 2.00	63.92 ± 6.29
**Lipid profile**				
Triacylglycerides (TAG) (mg/dL)	75.8 ± 13.9	63.3 ± 5.9	143.3 ± 14.6 **	97.7 ± 16.3
Total cholesterol (TC) (mg/dL)	50.7 ± 4.5	62.8 ± 1.7	57.2 ± 6.7	55.2 ± 5.3
High-density lipoproteins cholesterol (HDL) (mg/dL)	31.7 ± 2.8	39.2 ± 1.1	37.2 ± 4.9	36.5 ± 3.4
Very low-density lipoproteins cholesterol (VLDL) (mg/dL)	16.8 ± 3.5	12.5 ± 1.2	26.7 ± 3.4	19.5 ± 3.2
Low-density lipoproteins cholesterol (LDL) (mg/dL)	14.2 ± 1.7	17.2 ± 0.9	17.7 ± 3.1	15.5 ± 2.3
**Inflammatory markers**				
C-reactive protein (CRP) (mg/L)	0.10 ± 0.00	0.12 ± 0.02	0.12 ± 0.02	0.12 ± 0.02
Nitric oxide (NO) (uM)	5.2 ± 0.4	4.0 ± 0.5	9.6 ± 2.0	6.2 ± 1.2
N-acetylglucosaminidase (NAG) (nmol/mL)	280.8 ± 20.7	345.5 ± 22.7	546.5 ± 65.1 **	390.4 ± 69.6
**Electrolytes**				
Sodium (Na) (mmol/L)	148.7 ± 1.6	151.3 ± 0.6	146.5 ± 1.4	148.0 ± 0.5
Potassium (K) (mmol/L)	4.7 ± 0.3	4.8 ± 0.3	5.0 ± 0.2	4.9 ± 0.2
Chloride (Cl) (mmol/L)	99.7 ± 1.7	99.8 ± 0.4	97.8 ± 1.2	99.8 ± 0.7
Magnesium (Mg) (mEq/L)	1.7 ± 0.09	1.7 ± 0.07	1.8 ± 0.2	1.9 ± 0.1
Calcium (Ca) (mEq/L)	4.8 ± 0.2	4.6 ± 0.07	4.9 ± 0.1	4.8 ± 0.1
Phosphorus (P) (mg/dL)	7.3 ± 0.2	7.0 ± 0.2	7.1 ± 0.3	7.0 ± 0.4

Data are presented as means ± S.E.M; *n* = 6 per group. * *p* < 0.05, ** *p* < 0.01, *** *p* < 0.001, **** *p* < 0.001 significantly different from CONT; # *p* < 0.05, ## *p* < 0.01, ### *p* < 0.001 significantly different from FRUCT; + *p* < 0.05, ++ *p* < 0.01 significantly different from CP (by one-way ANOVA followed by Tukey’s post-hoc test. Abbreviations: CONT, control; CP, coffee pulp; FRUCT, fructose; HOMA-IR, homeostatic model assessment of insulin resistance; HOMA-ß, homeostatic model assessment of beta-cell function.

## Data Availability

Data are available on request from the authors.

## Supplementary Materials

The following supporting information can be downloaded at: https://www.mdpi.com/article/10.3390/antiox14030266/s1, Table S1: Chemical composition of feed and dried coffee pulp. Table S2: RT-PCR primers sequences and cycling conditions.

## Abbreviations

The following abbreviations are used in this manuscript:

ACC	Acyl-CoA carboxylase
ALP	Alkaline phosphatase
ALT	Alanine aminotransferase
AST	Aspartate aminotransferase
AT	Adipose tissue
BILID	Direct bilirubin
BILIT	Total bilirubin
BMI	Body mass index
CP	Coffee pulp
CRP	C-reactive protein
DBP	Diastolic blood pressure
FAS	Fatty acid synthase
G6P	Glucose-6-phosphate
GAPDH	Glyceraldehyde 3-phosphate dehydrogenase
GK	Glucokinase
GLU	Glucose
GLUT2	Glucose transporter 2
GLUT4	Glucose transporter 4
GS	Glycogen synthetase
GSSG	Oxidized glutathione
H&E	Hematoxylin and Eosin
HDL	High-density lipoprotein
HK2	Hexokinase II
HOMA-IR	Homeostasis model assessment for insulin resistance
HOMA-β	Homeostasis model assessment for β-cell function
ITT	Insulin tolerance test
LDL	Low-density lipoprotein
MASH	Metabolic dysfunction-associated steatohepatitis
MASLD	Metabolic dysfunction-associated steatotic liver disease
MAT	Mesenteric adipose tissue
MDA	Malondialdehyde
MetS	Metabolic syndrome
NAG	α-N-acetylglucosaminidase
NO	Nitric oxide
OGTT	Oral glucose tolerance test
ORO	Oil Red O
PAS	Periodic Acid-Schiff
Red GSH	Reduced glutathione
ROS	Reactive oxygen species
SBP	Systolic blood pressure
SCFAs	Short-chain fatty acids
SREBP-1c	Sterol regulatory element-binding protein 1
T2D	Type 2 diabetes
TC	Total cholesterol
TAG	Triacylglycerides
TBARS	Thiobarbituric acid reactive substances
Total GSH	Total glutathione
UA	Uric acid
VLDL	Very low-density lipoprotein cholesterol
WAT	White adipose tissue
XO	Xanthine oxidase
