# Supplementary material for: Sustainable Utilization of Coffee Pulp, a By-Product of Coffee Production: Effects on Metabolic Syndrome in Fructose-Fed Rats
Source: Antioxidants (Basel). 2025 Feb 25;14(3):266. doi: 10.3390/antiox14030266 (PMC11939298; doi:10.3390/antiox14030266)
Supplement: Supplementary file 1 [file antioxidants-14-00266-s001.zip › antioxidants-3485021-supplementary.pdf]

Supplementary Data

Supplementary Table S1. Chemical composition of feed and dried coffee pulp.

**Analysed parameters**	**Feed**	**Dried coffee pulp**
Moisture (g/100 g)	8.95 ± 0.40	12.07 ± 0.57
Ash (g/100 g)	5.94 ± 0.09	5.58 ± 0.32
Crude Protein (g/100 g)	19.15 ± 0.34	6.16 ± 0.17
Total Fat (g/100 g)	2.51 ± 0.01	0.78 ± 0.07
Total Carbohydrates (g/100 g)	63.44 ± 0.80	75.42 ± 0.41
Total Dietary Fibre (g/100 g)	19.67 ± 0.53	33.86 ± 0.14
Insoluble Dietary Fibre (g/100 g)	15.75 ± 1.14	27.87 ± 0.50
Soluble Dietary Fibre (g/100 g)	3.92 ± 1.67	5.99 ± 0.64
Remaining Carbohydrates (g/100 g)	41.55 ± 0.43	43.77 ± 1.16
Energy (kcal/100 g)	220.51 ± 3.70	244.15 ± 0.79
Energy (kJ/100 g)	923.38 ± 15.48	1017.42 ± 3.37
Fatty acids (relative %)		
C14:0	0.87 ± 0.04	1.22 ± 0.08
C16:0	15.31 ± 0.12	36.95 ± 1.56
C16:1	0.95 ± 0.03	n.d.
C18:0	2.89 ± 0.19	7.10 ± 0.62
C18:1*n9c*	25.36 ± 0.04	20.83 ± 4.97
C18:2*n6c*	47.93 ± 0.25	20.66 ± 1.72
C18:3*n*3	4.10 ± 0.05	13.25 ± 1.15
C20:1*n*9	0.77 ± 0.06	n.d.
C20:5*n*3	0.80 ± 0.01	n.d.
C22:6*n*3	1.01 ± 0.08	n.d.
Caffeine (g/100 g)	n.d.	0.50 ± 0.01
Chlorogenic acids (mg/100 g)		
3-caffeoylquinic acid	n.d.	2.17 ± 0.23
5-caffeoylquinic acid	n.d.	133.12 ± 2.26
4-caffeoylquinic acid	n.d.	6.24 ± 0.33

Abbreviations: n.d., not detected

Supplementary Table S2. RT-PCR primers sequences and cycling conditions.

	Primer sequence (5'- 3')	Annealing temperature (°C)	Cycles
HK2	Fwd: CAG CCT AGA CCA GAG CAT CC<br>Rev: CGC ATC TCT TCC ATG TAG CA	59	60
GS	Fwd: AGA AAT CAC AGC CAT CGA GGC<br>Rev: GTT CAA GCC GTT TGG AGT CAC	60	50
GK	Fwd: CAT ATG TGC TCC GCA GGA CTA G<br>Rev: CTT GTA CAC GGA GCC ATC CA	60	50
FAS	Fwd: GTG GGT CTC CTC CGA AGC CG<br>Rev: AGC ATG TCT TCG ATG TCG GTC AAG	60	40
ACC	Fwd: CCT TGT CAA CGC ATG GGC GG<br>Rev: GGC TTT GGG GTG GGG AGT CG	59	60
SREBP-1c	Fwd: GTG GGT CTC CTC CGA AGC CG<br>Rev: AGC ATG TCT TCG ATG TCG GTC AAG	60	40
GLUT2	Fwd: TTC TGT GCC GTC TTC ATG TC<br>Rev: TGG CCC AAT CTC AAA GAA AC	59	40
GLUT4	Fwd: GGC CGG GAC ACT ATA CCC<br>Rev: CCC CAT CTT CAG AGC CGA T	58	50
GAPDH	Fwd: GGC ATC GTG GAA GGG CTC ATG AC<br>Rev: ATG CCA GTG AGC TTC CCG TTA AGC	70	45

Abbreviations: HK2, hexokinase II; GS, glycogen synthetase; GK, glucokinase; FAS, fatty acid synthase; ACC, acyl-CoA carboxylase; SREBP-1c, sterol regulatory element-binding protein 1; GLUT2, glucose transporter 2; GLUT4, glucose transporter 4; GAPDH, glyceraldehyde-3-phosphate dehydrogenase.
